# High-order brain network feature extraction and classification method of first-episode schizophrenia: an EEG study

**DOI:** 10.3389/fnhum.2024.1452197

**Published:** 2024-10-23

**Authors:** Yanxia Kang, Jianghao Zhao, Yanli Zhao, Zilong Zhao, Yuan Dong, Manjie Zhang, Guimei Yin, Shuping Tan

**Affiliations:** ^1^Clinical Department, Beijing Huilongguan Hospital, Beijing, China; ^2^Laboratory of Brain Science and Intelligent Information Processing, School of Computer Science and Technology, Taiyuan Normal University, Jinzhong, China; ^3^Psychiatry Research Center, Beijing Huilongguan Hospital, Bejing, China; ^4^School of Chemical Engineering and Technology, Sun Yat-sen University, Zhuhai, China

**Keywords:** persistent homology, first-episode schizophrenia, high-order brain network features, random forest, light GBM

## Abstract

**Introduction:**

A multimodal persistent topological feature extraction and classification method is proposed to enhance the recognition accuracy of first-episode schizophrenia patients. This approach addresses the limitations of traditional higher-order brain network analyses that rely on single persistent features (e.g., persistent images).

**Methods:**

The study utilized resting-state EEG data from 198 subjects recruited at Huilongguan Hospital in Beijing, comprising 102 males and 96 females, with a mean age of 30 years and mean education of 14 years. Persistent topological features were extracted using adaptive thresholding during persistent homology (PH) filtrations. The distribution of these features was visualized through heatmaps and persistence entropies, while the generation process was elucidated using Betti curves and persistence landscapes.

**Results:**

The classification performance of the multimodal persistent topological features was assessed using various machine learning classifiers. The classifier yielding the highest performance was selected for comparison with traditional brain network features derived from graph theory and single persistent topological features. The results revealed significant topological changes in first-episode schizophrenia patients throughout the persistent homology filtering compared to healthy subjects. The univariate feature selection algorithm achieved a classification accuracy of 94.6% with a combination of attributes meeting the criterion of AC ≥ 0.6.

**Discussion:**

The proposed method demonstrates clinical significance for the early identification and diagnosis of first-episode schizophrenia patients, offering a new research perspective for constructing higher-order functional connectivity networks and extracting topological structure features.

## 1 Introduction

In the analysis of complex brain networks based on graph theory, different correlations between nodes and different metric thresholds lead to significant differences in brain network topology (Mammone et al., 2018). Some studies have shown that by integrating functional and structural neuroimaging and analysis, the unique information processing roles of synergistic and redundant components in the brain have been revealed, highlighting in particular the greater reliance on synergistic interactions by the human brain in support of higher-order cognitive functions (Luppi et al., 2022). Consequently, constructing complex brain networks based on connections between different functional regions has become a topic of discussion (Hasanzadeh et al., 2020). Traditional graph theory analysis methods typically involve manually setting a threshold for constructing brain networks (Morabito et al., 2015). However, this approach can result in an increased number of spurious connections or the omission of significant connections, leading to poorer generalization.

In recent years, topological data analysis (TDA) has emerged as a promising approach for analyzing functional brain networks (Ibáñez-Marcelo et al., 2019; Myers et al., 2019; Wang et al., 2019). Persistent homology (PH) plays a central role in TDA, as it allows comprehensive filtering of brain networks and analysis of structural changes, thereby identifying invariant features of their topology (Huber, 2021). For example, Spaziani (2019) used PH to extract topological features by analyzing brain networks across different sleep stages, enabling subject detection and classification. Existing studies using PH in brain network analysis primarily focus on exploring persistent graph features within a single modality. Entropy is widely used in higher order effects, and Stramaglia et al. use transfer entropy to parse the flow of information between dynamic processes in a network system (Stramaglia et al., 2024). Liu et al. (2021) used persistent entropy to summarize the survival distribution of midbrain node relations in persistent homology filtering of brain networks to analyze subjects’ perceived image quality. Chung et al. (2016) combined network analysis and heat kernel methods to classify functional brain networks using HCP task-based fMRI networks. Wang et al. (2021) used persistent landscape features to dynamically analyze the persistent homology filtering process in stroke patients, with particular emphasis on the evolutionary properties of the brain network filtering process. However, considering persistent features individually does not fully exploit the wealth of information present in the PH process, including multimodal information such as point distances, dynamic evolution, and the survival distribution of different persistent topologies (Zhang et al., 2021).

For this reason, this paper uses Pearson’s correlation (Benesty et al., 2009) to construct a brain network of resting-state EEG signals between patients with first-episode schizophrenia and healthy subjects. An adaptive thresholding method was used for PH filtering of the network. Heatmaps and persistent entropy were used to extract the filtered persistent feature distribution states. Simultaneously, Betti curves and persistent landscape features were computed from the persistent feature generation process states. Finally, multiple machine learning classifier models were used to evaluate the classification performance of the multimodal persistent features. The use of extracted persistent topological features of multimodal brain networks provides a more comprehensive understanding of changes in the global high-dimensional EEG data in first-episode schizophrenia patients and healthy subjects.

## 2 Methodology

### 2.1 Basic principles

**Figure 1:**
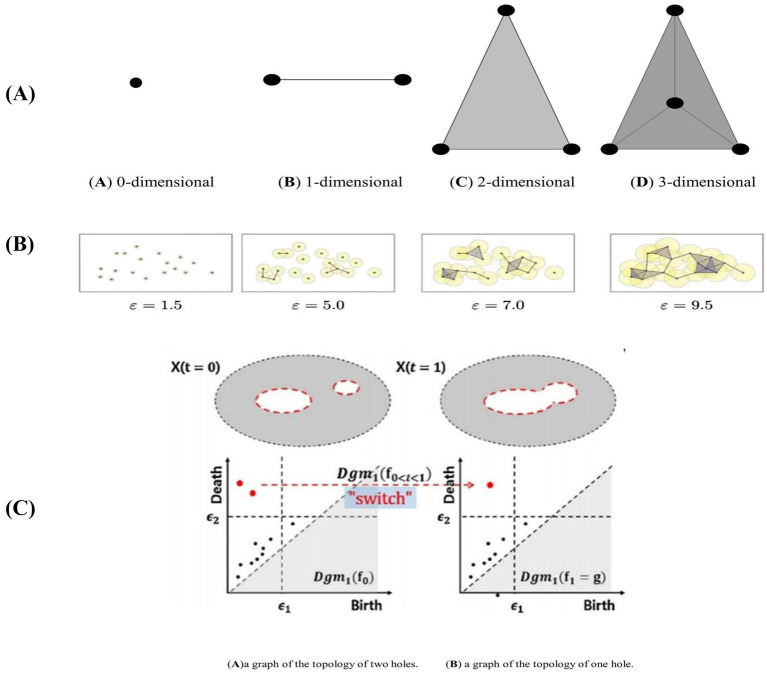
Complexes and topology changes in different dimensions in persistent homology. **(A)** Structural diagram representing the Vietoris-Rips (VR) complex in different dimensions. **(B)** represents the filtering process for point cloud data using different filtering thresholds **(Ca)** illustrates a graph with a topology consisting of two holes, represented by two two-dimensional persistence points on the persistence graph. **(Cb)** shows a graph with a topology of only one hole, resulting in a one-dimensional persistence point on the persistence graph.

PH captures persistently changing topological information in metric space through a persistent filtering approach. The specific filtering process is as follows: first, Vietoris-Rips (VR) complexes of different dimensions are constructed according to the specified threshold *ε*. When ε is larger than the metric between nodes in the network, a line is formed between two points (Sheehy, 2012), as illustrated in Figure 1A. This results in persistent topological features under different dimensions, such as 0-dimensional, 1-dimensional, and 2-dimensional Betti numbers. These features are typically visualized using a Persistence Diagram (PD) or barcode.

As *ε* gradually increases from 0, the connections in the network continuously change, and the number of VR complexes evolves until the VR complexes and connections in the PD reach a stable state, indicating that *ε* has reached its maximum. During the construction of VR complexes, some topologies persist for longer periods and represent the more significant topological features of the network. Conversely, other topologies exist only briefly and are overwritten as ε increases; these are considered perturbations or noise (Turkes et al., 2022). The topologies that persist longer indicate important topological relationships within the network and are the persistent topological features to be extracted. As ε changes, the network’s topological structure evolves, as illustrated in Figure 1B.

In the experiment, the generation threshold (b_i_) and disappearance threshold (d_i_) of each Vietoris-Rips (VR) complex in the PD were recorded for each dimension. This resulted in a set of points {bi, di, dim}, where dim represents the dimension, and the corresponding PD was derived from these points. Isomorphism between two persistence modules occurs if and only if the PDs of the two networks are identical. Figure 1C (a) illustrates a graph with a topology consisting of two holes, represented by two two-dimensional persistence points on the persistence graph. Conversely, Figure 1C (b) shows a graph with a topology of only one hole, resulting in a one-dimensional persistence point on the persistence graph (Otter et al., 2017).

### 2.2 Adjacency matrix

In the EEG time series signals, node pairs are measured using the Pearson correlation coefficient. This coefficient is then used to construct an adjacency matrix between the nodes. The Pearson correlation coefficient is calculated as described in Schober et al. (2018):

$$D_{ij}=1-|\frac{C_{ij}}{\sqrt{C_{ii}C_{jj}}}|$$

where $C_{ij}$ represents the covariance between different nodes, i and j denote the indices of different nodes in the EEG signal.

### 2.3 Topological features

The topological features (Betti numbers) extracted by PH cannot be directly used as inputs for machine classification algorithms. Therefore, four persistent features are introduced: persistence landscape, Betti curves, heat kernel, and persistence entropy. These features can be directly applied to machine learning classification algorithms.

#### 2.3.1 Persistence landscape

**Figure 2:**
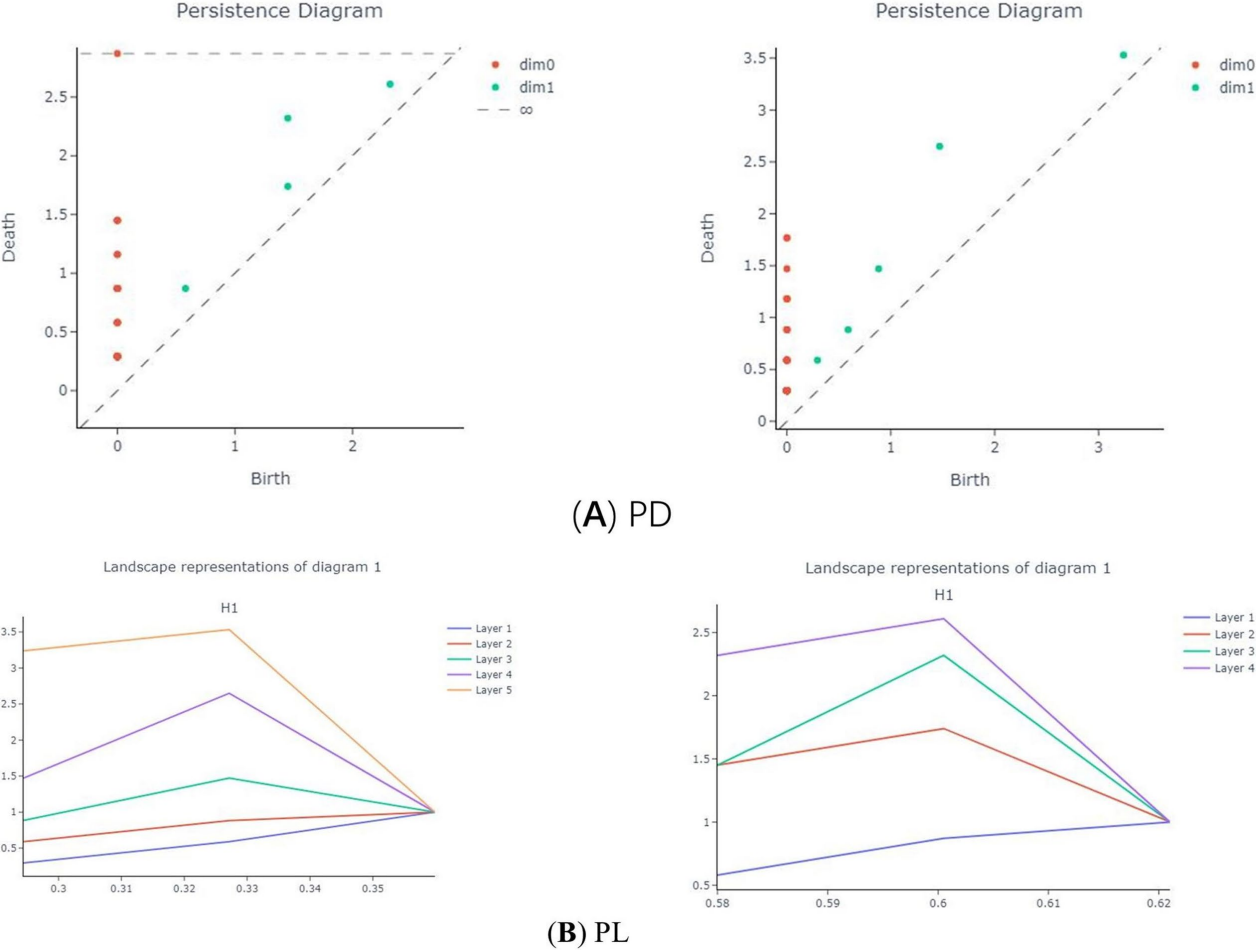

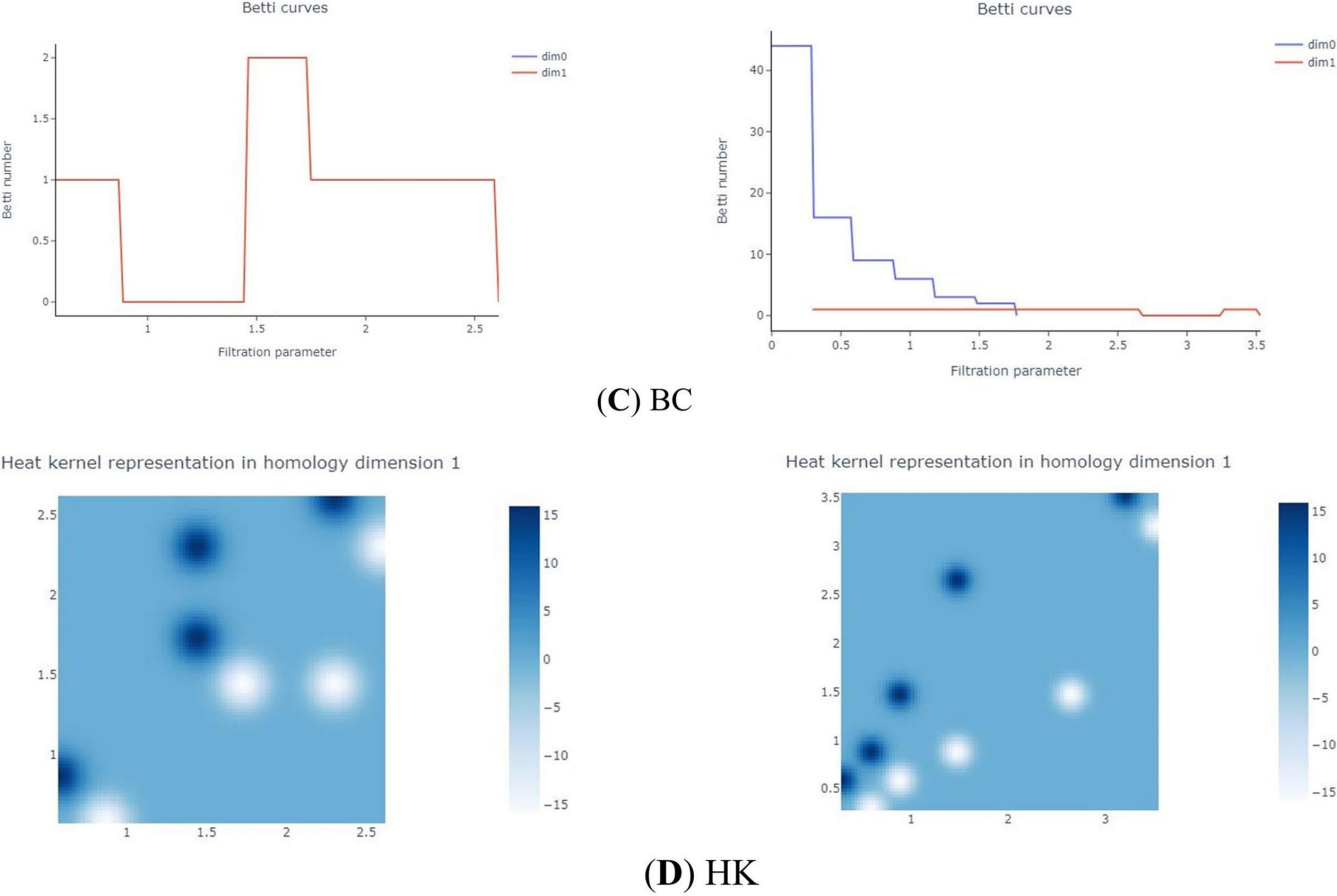
Patients (left) and normals (right) PD (A), PL (B), BC (C), HK (D).

Persistence Landscape (Bubenik, 2015) represents the persistence feature with a peak whose height is determined by the persistence of the feature, and its position corresponds to the feature’s position in the filtration. The conversion process from PD to PL is as follows:

First, the points in the PD are converted, and then a set of functions generated by the features of the rotated persistence image is considered to define the persistence landscape. The calculations are shown as follows. Given a point (𝑏_𝑖_,) in the PD, it is converted to $\left(\frac{b_{i}+d_{i}}{2},\frac{d_{i}-b_{i}}{2}\right)$ .

$$A_{i}\left(t\right)=\{\begin{array}{c} t-b_{i},t\in (b_{i},\frac{b_{i}+d_{i}}{2}]\\ d_{i}-t,t\in \left(\frac{b_{i}+d_{i}}{2},d_{i}\right)\\ 0,\mathrm{else} \end{array}$$

$$\left(t\right)=\max\left(A_{i}\left(t\right),0\right)$$

$$\gamma_{k}\left(t\right)=\mathrm{kmax}\left\{{\left\{\alpha_{i}\left(t\right)\right\}}_{i\in I}\right\}$$

where $A_{i}\left(t\right)$ , $A_{i}\left(t\right)$ is the diagonal set in the PD and $a_{i}\left(t\right)$ is the maximum value of $A_{i}\left(t\right)$ for each homology dimension *k* (Chukanov, 2023). Then, $\gamma_{k}\left(t\right)$ represents the persistence landscape of the *kth* homology dimension. Specifically, as shown in Figure 2, the plane is divided into multiple triangular regions i∈I at $\left(b_{i},d_{i}\right)$ intervals on the horizontal t-axis with a slope of 1. If $P_{k}$ is the intersection of the regions in the set of polygons I , then $\gamma_{k}$ is the tallest polygonal segment (the group of segments farthest from the t-axis) in the set $P_{k}$ .

#### 2.3.2 Betti curve

The Betti Curve (BC) (Laumon and Rapoport, 1995) represents the number of topological features in each dimension for each *ε* in the VR filtration. Specifically, the 0-dimensional BC denotes the number of connected components for each ε, while the 1-dimensional BC indicates the number of 1-dimensional holes for each ε. The transformation from PD to BC is illustrated in Figure 2.

#### 2.3.3 Heat kernels

Heat kernels (HK) are a multiscale convolution of PDs with Gaussian kernels (Reininghaus et al., 2015). It samples the $PDs={\left\{\left(d_{i}-b_{i}\right)\right\}}_{i\in I}$ of different homology dimensions as a sum of Dirac-Delta functions uniformly from the specified filtration parameters, converting the PDs into a matrix. Then, the convolution of the PDs is calculated using a Gaussian kernel. This process is also applied to the reflectance image of the PD diagonal, and the difference between the two convolutions is computed (Kulkarni et al., 2020), resulting in a multi-channel raster image, or Heatmap (HM), as shown in Figure 2.

#### 2.3.4 Persistence entropy

Persistence Entropy (PE) is the entropy of the persistence graph, calculated according to the definition of entropy (Rucco et al., 2016). The calculation is shown as:

$$E\left(PD\right)=-\underset{i\in I}{\sum }p_{i}\log\left(p_{i}\right)$$

where $p_{i}=\frac{\left(d_{i}-b_{i}\right)}{L_{D}}L_{PD}=\underset{i\in I}{\sum }\left(d_{i}-b_{i}\right)$ .

Persistent entropy can globally summarize the information about the topological structure due to its strong correlation with the topology.

## 3 Experiment

### 3.1 Persistent topological feature classification model for first-episode schizophrenia patients based on PH

**Figure 3:**
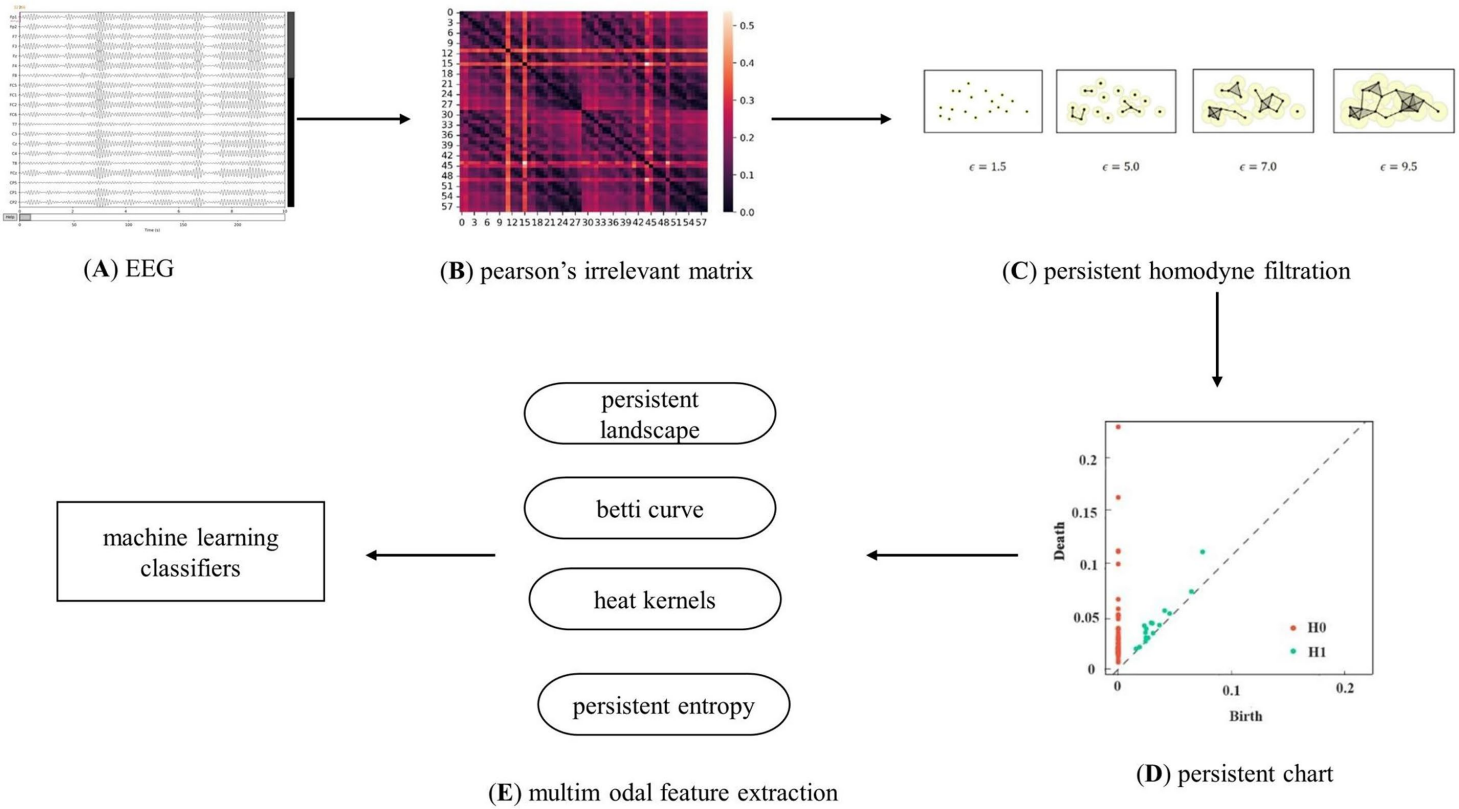
Analysis flow chart. Clarify the experimental flow, which is mainly divided into EEG data processing, brain network construction using Pearson’s correlation coefficient and V-R complex construction, topological feature extraction, and classification using machine learning algorithms.

Figure 3 shows the classification model of persistent topological features for patients with first-episode schizophrenia based on PH. Following the preprocessing of the resting-state EEG data, each EEG dataset was divided into five frequency bands. Subsequently, the adjacency matrix of 59 nodes for each subject in each frequency band was obtained. The network was constructed and filtered using the VR filtering algorithm to obtain the basic topological feature PD, which was then transformed into the multimodal topological features PL, BC, HK, and PE. Finally, four machine learning classifiers were used to verify the effectiveness of the feature classification.

### 3.2 Experimental data

The experiment used 59-channel EEG data collected from 104 first-episode schizophrenia patients and 94 healthy subjects in a closed-eye resting state at Huilongguan Hospital. By matching gender, age, and education level, demographic and clinical data, including PANSS scores, were compiled for the two groups, as shown in Table 1.

**Table 1:** Statistics of demographic and clinical data of subjects in two groups.

Features	Schizophrenic patients (*n* = 104)	Normal subjects (*n* = 94)	Statistical value
Average age (years)	30.49(20–50)	30.50(17–48)	F_1,196_ < 1
Time of education (years)	14.15(9–19)	14.73(9–19)	F_1,196_ = 2.697, *p* = 0.102
Gender (male/female)	52/52	50/44	χ_12_ = 0.201, *p* = 0.654
PANSS score	75.28 ± 11.10		
Positive score	21.77 ± 4.89		
Negative score	17.32 ± 5.84		

Data acquisition for this experiment was performed using a 64-lead EEG device from NeuroScan, Inc. The sampling frequency was 500 Hz, with impedance kept below 5 kΩ. The ground electrode was placed at AFz, and reference electrodes were physically attached to the left and right mastoids. Vertical electroencephalography involves placing electrodes above and below the left eye, while horizontal electroencephalography involves placing electrodes on the orbital rim of the right eye.

Data preprocessing was performed using EEGLAB. The reference electrodes were converted to a mean reference, and noise components were removed from each subject’s data. Independent Component Analysis (ICA) was used to remove ocular artifacts from the signal. After artifact removal, the Event-Related Potential (ERP) components were extracted by filtering, segmenting, and averaging the stacked trials.

### 3.3 Data preprocessing

In the experiment, the EEG signal data was preprocessed using Python’s MNE toolkit. The signals were filtered into five frequency bands: Delta (1–3 Hz), Theta (4–7 Hz), Alpha (8–12 Hz), Beta (13–30 Hz), and Gamma (31–49 Hz).

The duration of each sample was approximately 230 s. A sliding window (length = 40s, TR = 40s) was used to segment the data from 40 to 200 s to express the topological relationship of the brain. Repeated experiments were conducted on all EEG signals using non-overlapping sliding windows, and it was determined that the EEG signals from 126 to 150 s (Zhang et al., 2021) best expressed the topological relationship of the brain.

### 3.4 Construction of the adjacency matrix

**Figure 4:**
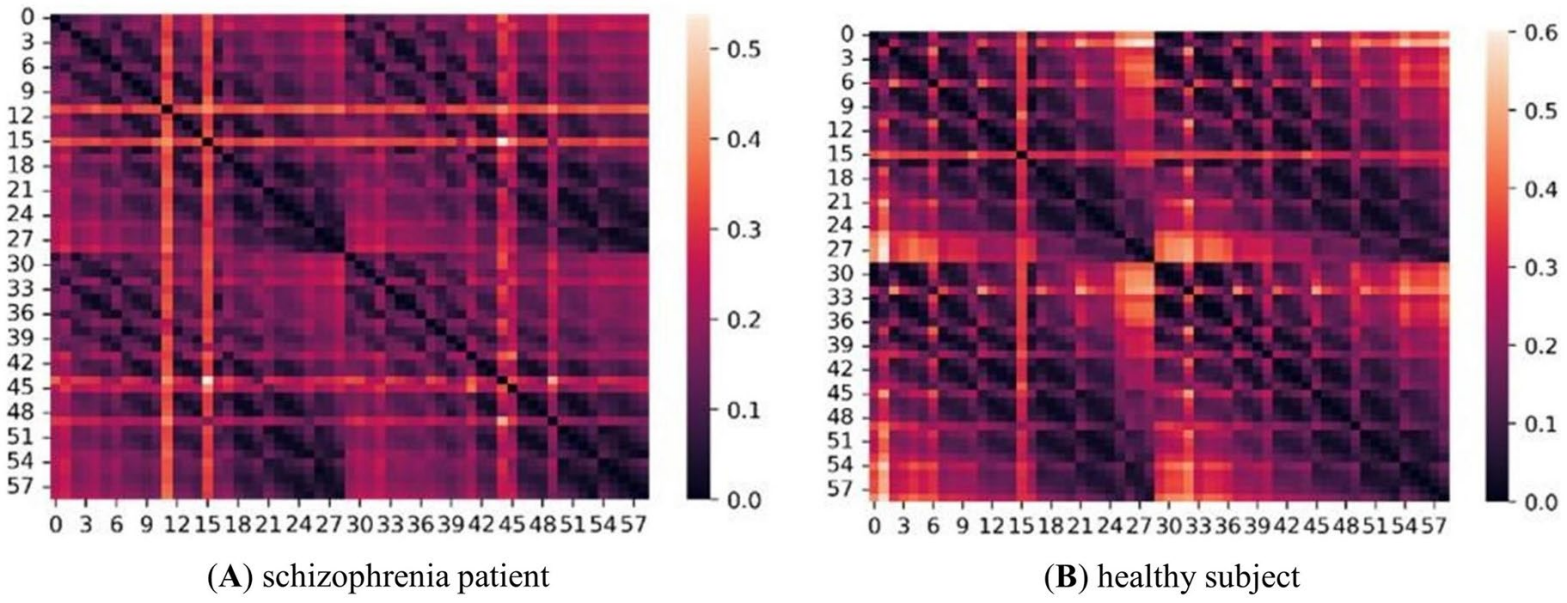
Adjacency matrix diagram of two groups of subjects in the *γ*-band. **(A)** represents schizophrenia patient and **(B)** represents healthy subject. The size is 59*59, and the right side is the scale label, the lighter color indicates the higher correlation between two two nodes.

The adjacency matrices of the five frequency bands for the two groups of subjects were constructed as described in section 1.1. Figure 4 shows an adjacency matrix plot for the gamma band.

### 3.5 Construction of the brain networks and VR complexes

**Figure 5:**
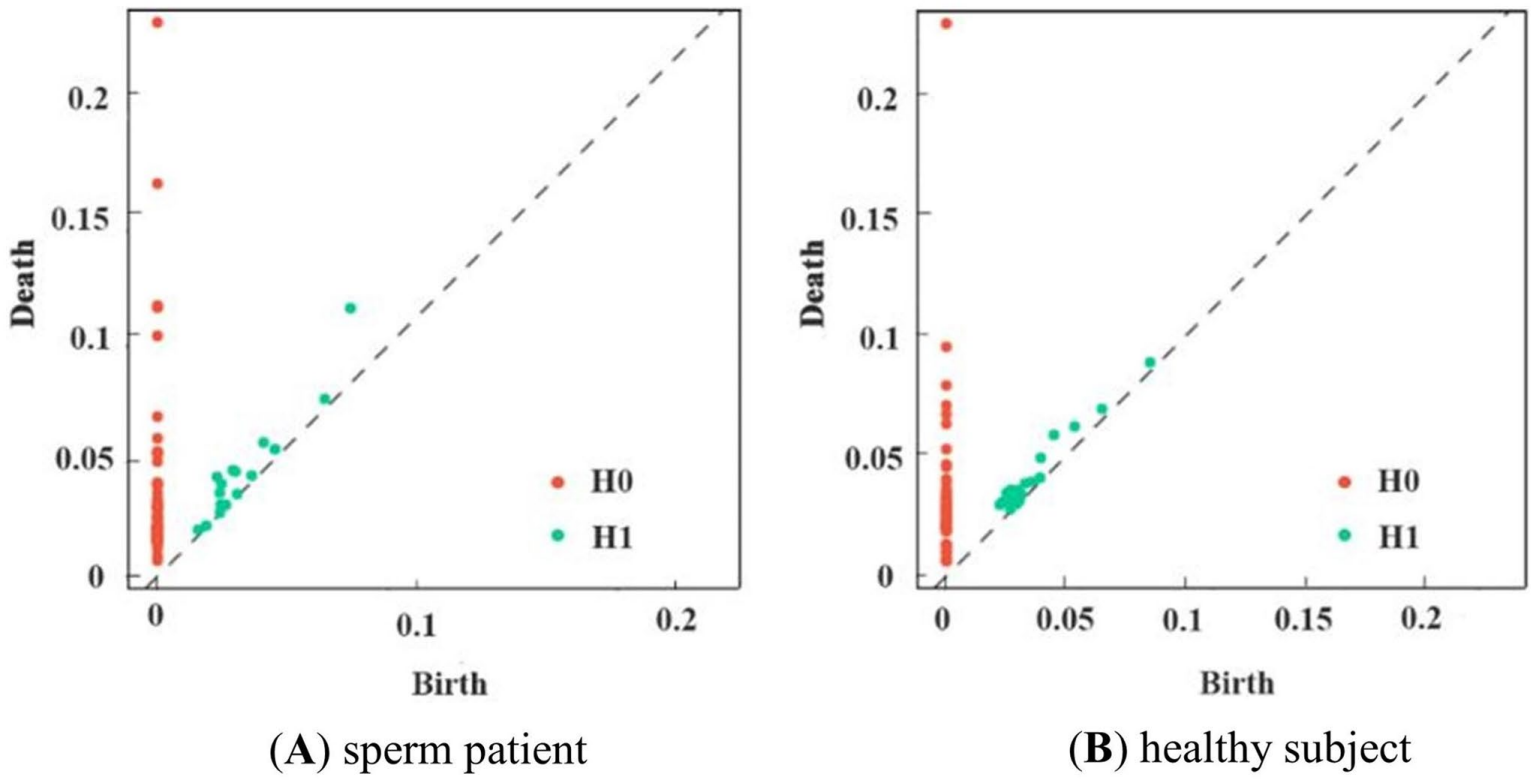
PD of subjects in the two groups in the *γ*-band. **(A)** shows schizophrenia patient, and **(B)** shows the healthy subject. Consisting of (birth, death, dim), red dots indicate 0-dimensional and green dots indicate 1-dimensional data.

Experiments were conducted using the Giotto-TDA Topology Machine Learning Toolkit in Python to build brain networks and VR complex shapes (Tauzin et al., 2021). In the model, the maximum threshold for VR filtering was not set as a fixed value. Instead, the construction of the brain network using VR filtering was concluded dynamically by determining when the number of VR complexes in the brain network ceased to change (Bauer, 2021). Topological features obtained through VR filtering were visually represented using PD. Figure 5 shows the PD of first-episode schizophrenia patients and healthy subjects in the Gamma band.

### 3.6 Topological feature extraction

Based on the extraction of the basic persistent feature PD, PD is converted into PL, BC, and HK according to the methods described in sections 1.2.1–1.2.3. These three features represent the features of PD from different modes. Compared to direct feature extraction from complex EEG data for classification, converting PD into multimodal features offers extremely high interpretability and visualization.

To summarize the three obtained topological features, PL, BC, and HK are summarized using matrix norms (1-norm and 2-norm), and the features are numbered. The corresponding number of features is shown in the table. A matrix norm is obtained for each dimension of the VR complex, yielding feature values that can be used for machine learning classification. Additionally, changes in the VR filter structure are an important representation of the topological features, so the PE corresponding to the PD is calculated. Tables 2, 3 show the selection of topological features and the corresponding parameters used in the experiment.

**Table 2:** Selection of topological feature parameters.

Topological features	Parameters selection
PL	1 layer 1 norm
	2 layers 1 norm
	1 layer 1 norm
	2 layers 1 norm
BC	1 norm
	2 norms
HK	Gaussian kernel standard deviation 1.6 1 norm
	Gaussian kernel standard deviation 3.2 1 norm
	Gaussian kernel standard deviation 1.6 2 norms
	Gaussian kernel standard deviation 3.2 2 norms
PE	none

**Table 3:** Topological feature numbers.

Number	Topological features
1	Landscape “p”: 1, “n_layers”: 1, “n_bins”: 100 H0
2	Landscape “p”: 1, “n_layers”: 1, “n_bins”: 100 H1
3	Landscape “p”: 1, “n_layers”: 2, “n_bins”: 100 H0
4	Landscape “p”: 1, “n_layers”: 2, “n_bins”: 100 H1
5	Landscape “p”: 2, “n_layers”: 1, “n_bins”: 100 H0
6	Landscape “p”: 2, “n_layers”: 1, “n_bins”: 100 H1
7	Landscape “p”: 2, “n_layers”: 2, “n_bins”: 100 H0
8	Landscape “p”: 2, “n_layers”: 2, “n_bins”: 100 H1
9	Betti “p”: 1, “n_bins”: 100 H0
10	Betti “p”: 1, “n_bins”: 100 H1
11	Betti “p”: 2, “n_bins”: 100 H0
12	Betti “p”: 2, “n_bins”: 100 H1
13	Heat “p”: 1, “sigma”: 1.6, “n_bins”: 100 H0
14	Heat “p”: 1, “sigma”: 1.6, “n_bins”: 100 H1
15	Heat “p”: 1, “sigma”: 3.2, “n_bins”: 100 H0
16	Heat “p”: 1, “sigma”: 3.2, “n_bins “:100 H1
17	Heat “p”: 2, “sigma”: 1.6, “n_bins”: 100 H0
18	Heat “p”: 2, “sigma”: 1.6, “n_bins”: 100 H1
19	Heat “p”: 2, “sigma”: 3.2, “n_bins”: 100 H0
20	Heat “p”: 2, “sigma”: 3.2, “n_bins”: 100 H1
21	Persistence Entropy H0
22	Persistence Entropy H1

## 4 Results

The experimental results are presented in three parts. The first part shows the PD performance of the two groups of subjects in each frequency band after VR filtering. The second part illustrates the machine learning classification performance of the multimodal persistent topological features. The third part details the classification accuracy and feature distribution of the two types of features.

### 4.1 Persistence image

**Figure 6:**
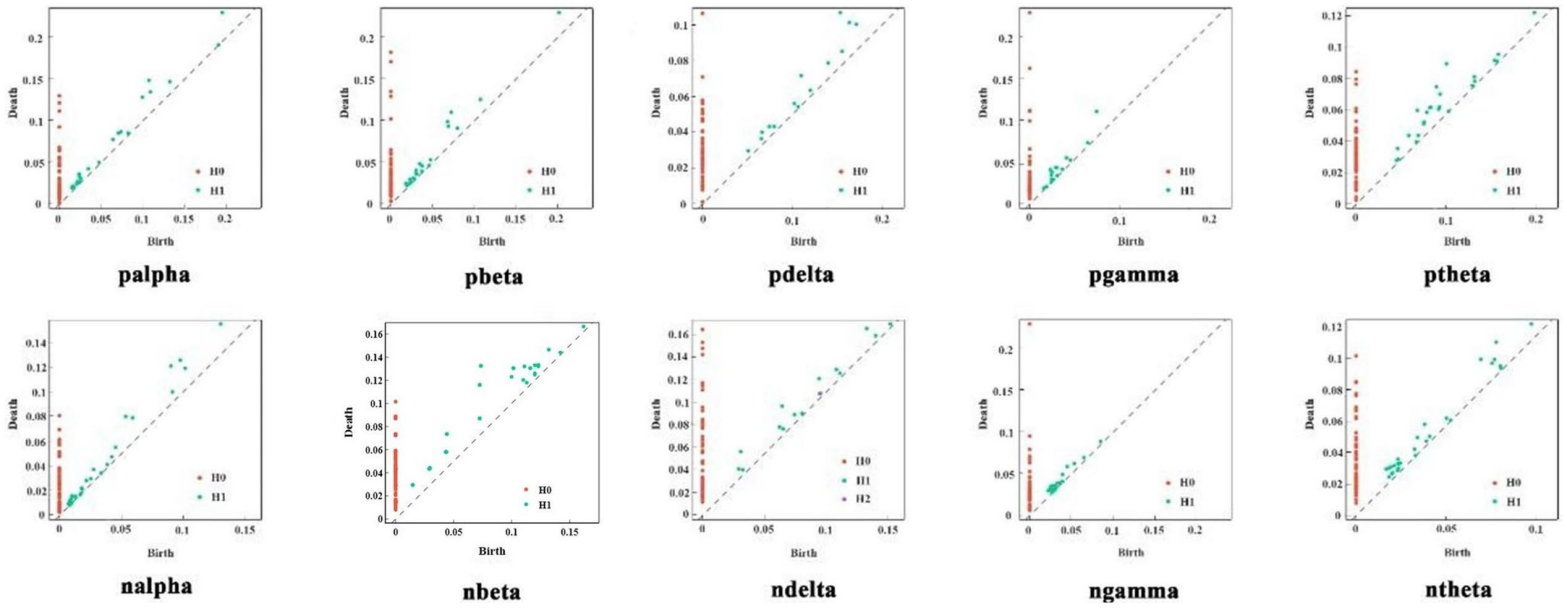
PD of two groups of subjects in five frequency bands.

In the experiment, persistent homology filtering was applied to the two groups of subjects across five frequency bands. The results are shown in Figure 6, which illustrates significant differences in the PD between healthy subjects and patients in each frequency band. In the alpha band, both patients and healthy subjects exhibited a large number of two-dimensional topologies clustered in the shorter threshold region, suggesting the presence of dense, unobservable holes. However, for more persistent two-dimensional topological features, the two-dimensional topology of the patients was tighter than that of the healthy subjects, with shorter edge relations. This trend was also observed in the Beta band, where the two-dimensional topology of healthy subjects was generally more clustered in the higher threshold portion of the distribution compared to the schizophrenia patients. The Delta and Gamma frequency bands showed similar performance. In the Theta frequency band, the two-dimensional topological distribution of healthy subjects was mainly concentrated at high-threshold and low-threshold positions, while for patients, it was mainly concentrated at mid-threshold positions. Overall, the topology of patients was more persistently homotopic relative to healthy subjects. These observations indicate that the distribution of topological features between patients and healthy subjects is distinct and regular, suggesting that these features have learnable properties.

### 4.2 Machine learning classification effect

The extracted topological features of each subject in each frequency band were combined to form the training data. This resulted in n subjects generating 5 (frequency bands) *N pieces of data, with the feature dimension of each piece of data being m (number of extracted features) + 1 (frequency band sequence number). For the extracted data, we used four machine learning classification models (LR, SVM, Random Forest, Light GBM) for classification and evaluated feature performance using accuracy. Additionally, we compared the global and local features (modularity, local efficiency, clustering coefficient, and characteristic path length) of the brain network based on traditional graph theory and a single persistent feature in a multi-machine learning classification model. The results, shown in Table 4 using fivefold cross-validation, indicate that the accuracy of multimodal persistent features is higher. Based on the results in Table 4, we used the Light GBM classifier, which had the highest accuracy, to compare different features using multiple indices. Under the four indices of accuracy, precision, recall, and F1 score, multimodal persistent features demonstrated better performance. The results are shown in Table 5.

**Table 4:** Comparison of the classification performance of multiple classifiers with different features.

Features	LR	SVM	RF	Light GBM
Diagram properties	0.703	0.678	0.722	0.739
BC	0.738	0.741	0.752	0.755
PE	0.697	0.721	0.749	0.744
PL	0.655	0.706	0.721	0.734
HM	0.823	0.811	0.817	0.841
BC + PE + PL + HK	0.879	0.854	0.866	0.913

**Table 5:** Comparison of the classification effects of different features.

Features	Accuracy	Precision	Recall	F1
Diagram properties	0.739	0.720	0.825	0.769
BC	0.755	0.731	0.844	0.783
PE	0.744	0.767	0.737	0.752
PL	0.734	0.831	0.621	0.711
HM	0.841	0.839	0.864	0.851
BC + PE + PL + HK	0.897	0.887	0.922	0.904

### 4.3 Persistent topological feature distribution

**Figure 7:**
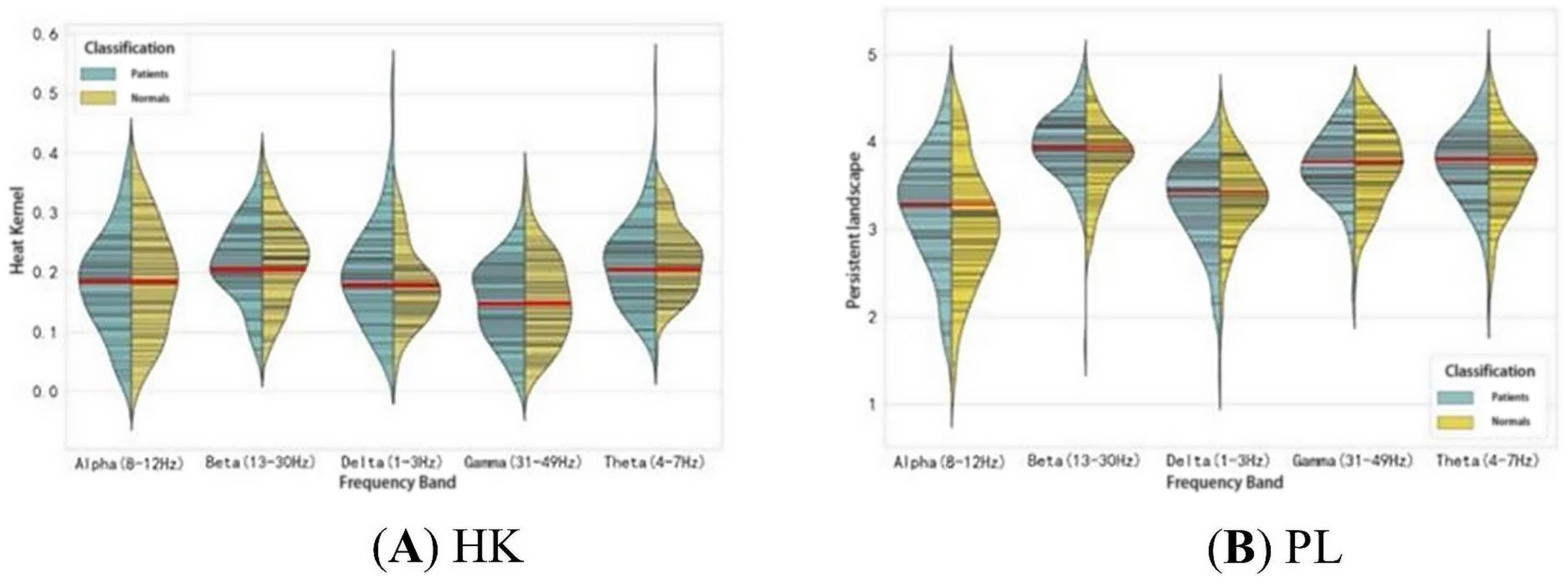
Feature distributions of HK (Gaussian kernel standard deviation 3.2, 1-norm) and PL (1-norm) for the two groups of subjects in the five frequency bands.

As seen in Table 4, the HK, PE, and PL (Gaussian kernel standard deviation 3.2, 1-parameter) features in the Light GBM boosted tree model are significantly effective in identifying first-episode schizophrenia patients. These features focus on different aspects of the persistent topological features. HK primarily addresses the threshold distribution of PD, specifically the birth and death times of the persistent topological features, while PL emphasizes changes in the persistent topological features during filtration and the magnitude of these changes. The distributions of these two features are shown in Figure 7. There are significant differences in the distribution of HK and PL features between patients and healthy subjects across different frequency bands. In the Delta and Theta bands, the distribution of HK values in patients is more scattered and inconsistent compared to healthy subjects. In the Alpha band, the distribution of PL values shows a significant difference between patients and healthy subjects, suggesting that patients exhibit more variability throughout the persistent homology filter, whereas healthy subjects display more stability. This trend is also observed to varying degrees in the other frequency bands.

### 4.4 Single feature classification accuracy

The topological features extracted from the experimental data (with a training set consisting of 73 psychotic subjects and 60 normal subjects, and a test set of 30 psychotic subjects and 30 normal subjects) were selected using a univariate feature selection algorithm. The accuracy of single-attribute classification is shown in Table 6. Attributes with good performance (AC ≥ 0.55) or (AC ≥ 0.60) were selected. These attributes were then used for machine learning classification to assess classification performance, with the accuracy results shown in Table 7. After selecting the attribute combination, those with AC ≥ 0.6 were used as the input for the classifier. Grid parameter adjustment was performed to select appropriate model parameters. After optimizing the model, tenfold cross-validation was conducted, and the cross-validated classification results were averaged (Kohavi, 1995). The classification accuracies are shown in Table 7.

**Table 6:** Single feature classification accuracy.

Feature number	Alpha	Beta	Delta	Gamma	Theta
1	0.62	0.62	0.50	0.55	0.51
2	0.55	0.46	0.41	0.60	0.57
3	0.58	0.58	0.67	0.62	0.58
4	0.64	0.64	0.48	0.50	0.62
5	0.50	0.51	0.58	0.66	0.48
6	0.58	0.50	0.57	0.60	0.37
7	0.58	0.60	0.67	0.60	0.58
8	0.60	0.57	0.46	0.58	0.51
9	0.57	0.48	0.51	0.53	0.51
10	0.57	0.55	0.42	0.55	0.55
11	0.62	0.58	0.64	0.58	0.44
12	0.44	0.55	0.60	0.64	0.53
13	0.48	0.57	0.57	0.42	0.48
14	0.50	0.39	0.51	0.60	0.46
15	0.60	0.75	0.69	0.75	0.58
16	0.39	0.55	0.66	0.50	0.55
17	0.58	0.78	0.62	0.73	0.64
18	0.58	0.53	0.60	0.58	0.60
19	0.57	0.62	0.62	0.66	0.62
20	0.53	0.51	0.60	0.64	0.50
21	0.58	0.67	0.76	0.75	0.69
22	0.46	0.57	0.57	0.48	0.55

**Table 7:** Accuracy summary tables for feature combination and model optimization comparison.

	Alpha	Beta	Delta	Gamma	Theta
All properties	0.645	0.756	0.632	0.782	0.713
Single feature combination with accuracy AC ≥0.55	0.696	0.875	0.821	0.854	0.821
Single attribute ac ≥ combination of features for 0.60	0.729	0.729	0.875	0.821	0.854
Classification accuracy before optimization	0.729	0.875	0.854	0.875	0.854
Classification accuracy after optimization	0.875	0.908	0.946	0.908	0.925

## 5 Statistical results and analysis

A loop is the simplest structure that introduces structural redundancy and feedback dynamics into a network. Loops are prevalent in network research and, along with star structures, link structures, and others, are considered fundamental components of networks, especially complex networks (Boccaletti et al., 2006). It has been shown that networks designed based on loop structures have optimal synchronization ability (fully homogeneous networks) and control robustness. Additionally, loop structures are used to characterize the degree of local node aggregation within the network and to measure the degree of approximation between the network and tree networks.

The circle structure brings structural redundancy and feedback dynamics to a network. Studies indicate that networks designed based on circle structures have optimal synchronization capability (fully chiral networks) and control robustness. Additionally, circle structures are used to depict the degree of node clustering locally in the network and to measure the proximity between the network and tree networks.

The importance or role of loops in a network can be measured by the circle ratio (Ebert et al., 2016). The circle ratio is a new metric for ranking the importance of nodes. Comparing it with existing metrics reveals that the ranking results for important nodes identified by the circle ratio differ significantly from those identified by traditional metrics. When a complex network is attacked maliciously using the important nodes identified by the circle ratio, the network collapses faster. Alternatively, the network can reach a synchronized state faster by controlling these important nodes. This analysis suggests that the circle ratio is an effective alternative measure of node importance in complex networks.

The circle ratio refers to the extent to which a node participates in the shortest circles of other nodes. The shortest circle is the minimum length loop that contains this node. Traditional node centrality metrics focus on the node itself, considering the contributions of neighboring nodes. However, the circle ratio reverses this perspective, emphasizing how much a node contributes to its neighbors’ structural and dynamical processes. A node’s importance is determined by its participation in the neighborhood’s social roles (the number of circles containing it). This shift in perspective implies a philosophical change in assessing node importance (Zhao et al., 2020).

In each frequency band (five bands), each subject’s data is divided into four 40-s segments. After filtering the data, the results are presented as persistence graphs, where each point represents a homology group existing for a duration (excluding infinite values). The top five homology groups by duration are identified. Based on the birth and death times of each homology group, the corresponding two-dimensional simplices are identified. The points and edges of these simplices form a graph, from which each node’s circle ratio value is obtained. The top 10 nodes by circle ratio are recorded for each graph, resulting in 20 graphs per subject. The important nodes in the Delta band are shown in Table 8, and those in the Theta band are shown in Table 9.

**Table 8:** The number of times the patient’s Delta band critical node was recorded.

Node number	Number of times recorded
38	904
9	853
39	798
…	…
11	7
49	5
15	4

**Table 9:** The number of times the patient’s Theta band critical node was recorded.

Node number	Number of times recorded
9	975
8	925
38	874
…	…
45	12
1	5
11	3

**Figure 8:**
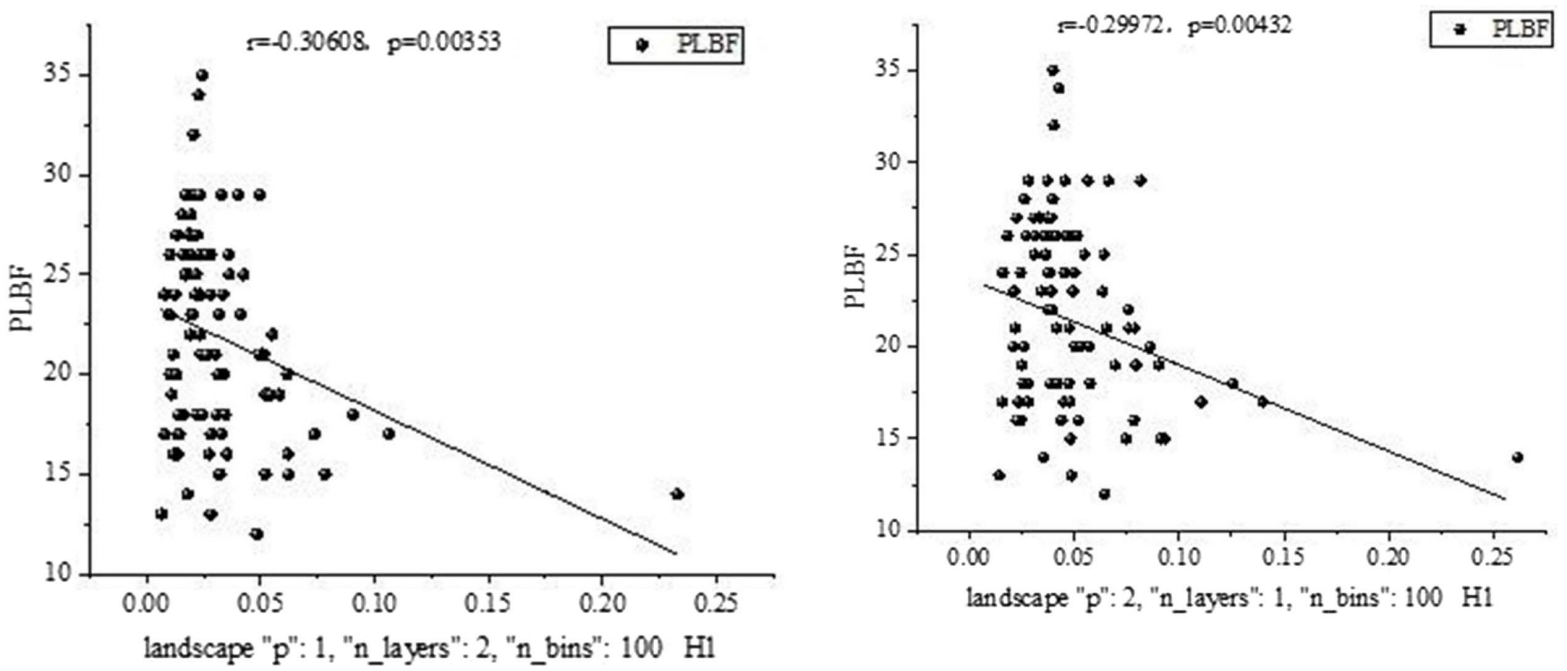
Some salient features and clinical measurements in the Delta band.

Statistical analysis was performed for all five bands, revealing a significant relationship between certain salient features and clinical measurements in the delta band, as shown in Figure 8.

**Figure 9:**
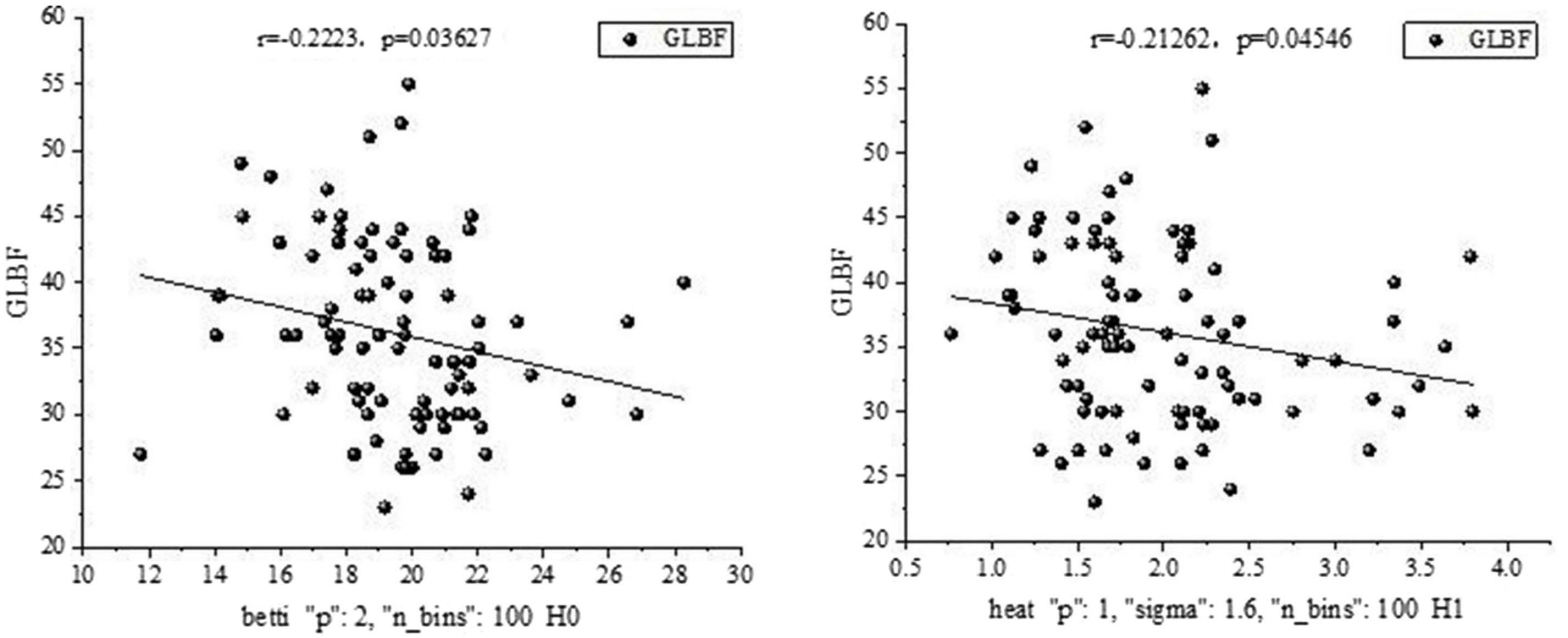
Some salient features and clinical measurements in the Theta band.

A significant relationship was found between certain salient features and clinical measures in the theta band, as shown in Figure 9.

## 6 Discussion

With the increasing popularity of complex networks, substantial advancements have been achieved in applying graph theory to brain networks. Utilizing traditional graph theory methods to extract features from brain networks for tasks like classification and prediction can significantly contribute to our comprehension of the pathogenesis and principles underlying schizophrenia. However, existing methods for disease classification are constrained by their focus solely on low-order graph features, thus posing challenges in capturing the diverse topological information inherent in the data (De Miras et al., 2023).

Persistent homology methods can play an important role in the extraction of topological information, but their potential in the construction of brain networks has not been fully exploited (Chung et al., 2024). Given the shortcomings of existing models, we propose a new method to extract multimodal persistent topological features from the complex brain networks of first-episode schizophrenia patients and improve their classification accuracy. By applying persistent homology and topological data analysis methods, we successfully extracted multimodal persistent topological features from resting-state EEG signal data and described and analyzed these features using tools such as heat maps, persistent entropy, Betti curves, and persistent landscape features. The experimental results show that our extracted multimodal persistent topological features comprehensively reflect the global topological changes in the high-dimensional brain network between first-episode schizophrenia patients and healthy subjects. In the persistent homology filtering, there is a significant difference between the PDs of healthy subjects and patients in each frequency band, and the overall topological change trend of the high-dimensional brain networks of healthy subjects and patients is found by observing the PD images in each frequency band. While the distribution of 2D topology of healthy subjects was more clustered in higher or lower regions in each frequency band, the overall topology of patients had more persistent homology relative to healthy subjects, showing significant regularity.

Compared to traditional graph theory-based methods and single persistent topological features (Yin et al., 2023), our method significantly improves classification accuracy, classification accuracy of 94.6% based on extracted multimodal persistent topological features. Additionally, through data filtering and persistence graph analysis methods, we transformed the raw data into persistence graphs and extracted the top five homology clusters. Based on the information from the homology clusters, we constructed the graph structure and calculated the circle ratio value of each node, using this node importance ranking metric to identify the important nodes in patients and normal subjects. The results showed that the important roles of the channels at the electrode positions of P4 and Fp1 in the subjects were the occipital lobe region of the right hemisphere of the brain and the anterior center of the brain, respectively, and that the important nodes of the subjects were changed from the occipital lobe region of the left hemisphere of the brain to the anterior center of the brain after the lesion, and that the changes in the brain regions of the subjects after the lesion indicated that the patients with schizophrenia had different degrees of changes in their perceptual and cognitive functions (Howes et al., 2023).

This study provides important clinical guidance for the early detection and diagnosis of patients with first-episode schizophrenia. By gaining a deeper understanding of the topological features of brain networks, we can better understand the functional brain connectivity of schizophrenic patients, and the proposed method can also be applied to other clinical areas, such as the use of higher-order networks to capture topological information in the construction of brain networks in Alzheimer’s disease with mild cognitive impairment (Hao et al., 2024; Huo et al., 2024), which has been further improved in feature extraction, and the integration of artificial intelligence with practical applications, which can be used to provide the basis for personalized treatment and intervention (Marshall et al., 2024).

## 7 Conclusion

In this paper, we propose a multimodal persistent topological feature extraction and classification method to address the problem that the use of single-modal persistent features in the analysis of brain networks based on persistent homology fails to fully exploit the rich topological information generated during the persistent homology filtering process. Based on the resting EEG data of the subjects, Pearson correlation was used to build brain networks and high-dimensional, globally effective features extracted from PH filtering were used for research classification.

Future studies can further explore the application of multimodal persistent topological features in other psychiatric disorders, combining them with other neuroimaging data sources such as functional magnetic resonance imaging (fMRI) and magnetoencephalography (MEG), to more comprehensively reveal the complexity of brain networks. Additionally, further optimization of classifier models and validation methods is an important direction for future research to improve classification accuracy and stability.

## Data Availability

The raw data supporting the conclusions of this article will be made available by the authors, without undue reservation.
